# Immunohistochemical features of a papillary squamous cell carcinoma of the endometrium with transitional cell differentiation

**DOI:** 10.1186/1746-1596-2-26

**Published:** 2007-07-23

**Authors:** Alfredo Ribeiro-Silva

**Affiliations:** 1 Department of Pathology, Ribeirão Preto Medical School, University of São Paulo, Brazil

## Abstract

An 84-year-old woman underwent hysterectomy due to a friable endometrial mass infiltrating almost half way through the myometrial wall. The tumor consisted of papillary structures with thin fibrovascular cores covered by several layers of pleomorphic cells. The deeply located neoplastic cells were ovoid with a pale eosinophilic cytoplasm resembling urothelial cells. A diagnosis of papillary squamous cell carcinoma of the endometrium with transitional cell differentiation was made. Although she recovered well after surgery, she died one year later because of disseminated disease. In an attempt to obtain new insights into the physiopathology of this very rare tumor, an immunohistochemical panel with 32 markers was performed. The neoplastic cells were positive for cytokeratin 5, vimentin, p63, p21, VEGF, Ki67, BAG1, and bcl-2. The expression of BAG-1 and bcl-2 may suggest that anti-apoptotic stimuli are preponderant in this neoplasm.

## Background

Primary squamous cell carcinoma (SCC) of the endometrium is uncommon, with only about seventy cases reported in the literature [1]. Its histological appearance is identical to that of SCC of the uterine cervix [1]. A transitional cell carcinoma of the endometrium is a carcinoma in which 90% of more of the neoplastic cells resemble the urothelial transitional cells. Lower transitional cell differentiation qualifies the tumor as a mixed carcinoma with transitional cell differentiation [2,3]. Transitional cell differentiation in endometrial carcinomas is extremely uncommon, with fewer than 15 cases reported [2,3]. In an attempt to obtain new insights into the physiopathology of this very rare tumor, an immunohistochemical panel was applied to a papillary SCC of the endometrium with transitional cell differentiation.

## Case presentation

An 84-year-old woman gravida 7 para 4, whose last normal menstrual period was 35 years ago, presented with postmenopausal bleeding and pelvic pain of 2 months duration. She denied any use of estrogen replacement therapy or any history of previous abnormal vaginal bleeding. Past medical history showed arterial hypertension under medical control. She had no previous history of lower urinary tract cancer. Her general physical examination was within normal limits. The pelvic examination, however, showed that the uterus was slightly enlarged for her age. The ovaries were not palpable. Gross examination of the cervix did not show any lesion.

An endometrial biopsy was performed and the diagnosis of SCC was made. A preoperative bone scan and a chest radiograph were normal. She underwent abdominal surgery consisting of total hysterectomy with bilateral salpingo-oophorectomy. There was no grossly apparent tumor on exploration of the abdomen. Pelvic washing was also performed, with cytological examination being negative for neoplastic cells.

The patient recovered well after surgery; however she died one year later due to generalized metastasis. An autopsy was not performed.

## Pathological findings

The resected uterus was irregularly enlarged and weighed 120 g. The endometrial cavity showed a 5 × 3 × 3 cm friable mass infiltrating almost half way through the myometrial wall. The surgical specimen was fixed in 4% formalin and sequential 3 mm sections were obtained throughout the specimen. All sections obtained from the tumoral mass were embedded in paraffin and 4 μm histological sections obtained from paraffin-embedded blocks were stained with hematoxylin-eosin. For immunohistochemical staining the paraffin-embedded blocks were cut into 3 μm sections, deparaffinized, and rehydrated. A standard avidin-biotin-peroxidase method was used (Novostain Super ABC kit, Novocastra, Newcastle upon Tyne, UK). The antibodies and the dilutions are specified in Table 1.

**Figure 1:**
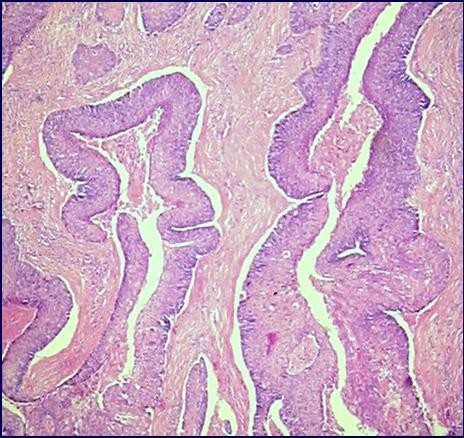
Squamous cell component showing fibrovascular cores covered with several layers of neoplastic cells (hematoxylin and eosin, ×200).

**Figure 2:**
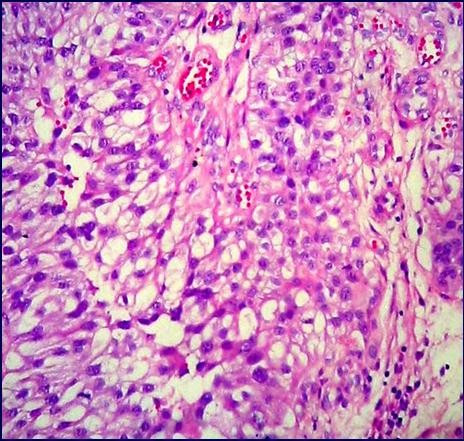
Transitional cell-like component of the tumor showing neoplastic cells with pale eosinophilic cytoplasm (hematoxylin and eosin, ×400).

Slides stained with hematoxylin-eosin showed a tumor consisting of papillary structures with thin fibrovascular cores covered by several cell layers with large amounts of cytoplasm and pleomorphic and hypercromatic nuclei (Fig. 1). The nucleoli were easily visualized. Mitoses were scarce. Keratinization was inconspicuous. The invasive front of the tumor was of the pushing type and the deeply located neoplastic cells were ovoid with a pale eosinophilic cytoplasm, resembling urothelial cells (Fig. 2). These cells represented 15% of the total tumor volume. A diagnosis of papillary squamous cell carcinoma with foci of transitional cell differentiation was made. The adjacent non-neoplastic endometrium exhibited an intense xanthogranulomatous reaction. There was an abrupt change between the tumor and the endometrium. There were also sparse foci of vascular lymph space invasion. The tumor did not reach the cervix or the adnexa which were entirely examined microscopically.

**Figure 3:**
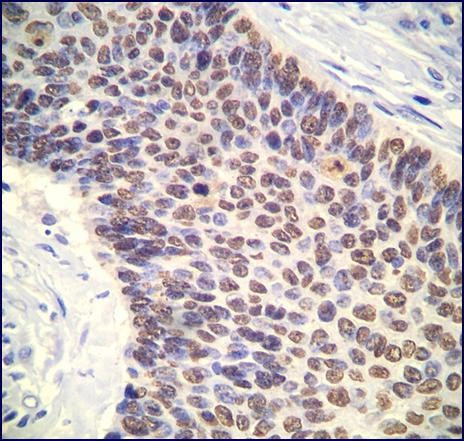
P63 expression (nuclear staining) throughout the thickness of the neoplastic epithelium (immunohistochemistry, ×200).

The immunohistochemistry study showed that more than 10% of the neoplastic cells were positive for BAG1, p21, VEGF and vimentin. The basal cells of the neoplastic epithelium stained diffusely for bcl-2 (80% of the cells). Ki67 stained 15% of neoplastic cells. Contrary to normal squamous tissue used as positive control, in which p63 stained only the basal cells, the squamous component of the carcinoma stained diffusely for p63 throughout the thickness of the epithelium (more than 90% of the cells) (Fig. 3). Cytokeratin 5 was diffusely positive in the squamous component and negative in the transitional cell areas. On the other hand, cytokeratins 8/18 were focally positive in the transitional cells (30% of the cells) but negative in the squamous areas. Both cytokeratins 7 and 20 were negative. The nuclei were diffusely positive for Chk2 and p27, which is considered to be the normal staining pattern for these markers. The other markers were negative.

## Discussion

An extension into the endometrium from a primary SCC of the cervix must be ruled out before making a diagnosis of pure SSC of the endometrium because this situation is far more common [4]. Besides, considering that pure squamous cell carcinoma of the endometrium is rare and a mixed endometrioid component may be focal, the tumor was entirely examined microscopically to rule out endometrioid carcinoma with extensive squamotransitional cell differentiation. The present case satisfied the three major criteria for the diagnosis of primary SCC of the endometrium: no glandular component was found after extensive sampling, the cervix was ruled out as the primary site, and there was no connection between the tumor and the stratified squamous epithelium of the cervix [4]. As observed in the present case, SCC of the endometrium usually occurs in postmenopausal women. Most patients undergo total abdominal hysterectomy and bilateral salpingo-oophorectomy followed or not by adjuvant radiation therapy [4]. The prognosis is poor, with the overall survival ranging from 3 to 42 months [4]. The patient of this report died 12 months after the surgery because of disseminated disease.

The diagnosis of transitional cell differentiation is made based on morphological rather than immunohistochemical features [2]. The morphological criteria include neoplastic cells with a pale eosinophilic cytoplasm resembling bladder urothelial epithelium [2]. Tumors with these features are more aggressive than conventional endometrial carcinomas [4]. All endometrial carcinomas with transitional cell differentiation are negative for cytokeratin 20 (CK20), but half are positive for cytokeratin 7 (CK7) [3]. This immunoprofile supports mullerian rather than urothelial origin. Some authors have noticed a similarity between the papillary variant of SCC and the transitional cell carcinoma of urothelial origin, but the exact relationship between them has not been fully elucidated [5]. In the present study the neoplasm was positive for cytokeratin 5 and negative for both cytokeratins 7 and 20. These findings agree with data reported by Stefansson et al. (2005) who verified that CK5/6 expression is more frequent in endometrioid tumors with squamous differentiation [6].

P63 is a marker of basal and squamous differentiation in several normal epithelia and human tumors and is considered to be a marker of progenitor or stem cells in stratified epithelia [6]. Contrary to normal squamous tissue used as positive control, in which p63 stained only the basal cells, the squamous component of the carcinoma stained diffusely for p63 throughout the thickness of the epithelium. In addition, there is increasing evidence that p63 may act as an oncogene in the tumorigenesis of endometrial carcinomas and its expression is significantly associated with high histologic grade, higher mitotic count and tumor cell proliferation, Ki-67 expression, microsatellite instability (MSI) and loss of hMSH6 expression [6]. These findings may suggest that the present tumor arose from undifferentiated stem cells, a hypothesis possibly explaining why a pure SCC developed in the endometrium. This hypothesis, although attractive, is merely speculative since p63 is frequently seen in various tumors showing squamous differentiation. In that way, the p63 immunohistochemical findings in this case may possibly only indicate that the tumor is of squamous differentiation rather than implying cell of origin.

In the present report, the carcinoma's proliferative rate was 15%, as assessed by the Ki67 labeling index. Ki67, however, is not considered a prognostic marker in endometrial carcinomas [7]. The neoplastic cells were positive for vimentin, p21, and VEGF. Vimentin expression was expected since it has also been consistently demonstrated in endometrial adenocarcinomas [8]. The expression of p21 and VEGF in endometrial carcinomas predicts myometrial invasion and lymph node metastasis and may prospectively identify patients who are at increased risk for poor outcome [9,10]. Indeed, in the present report, the patient had an unfavorable outcome. The tumor suppressor p27 was not down-regulated in the present case. In endometrial carcinomas, loss of p27 expression may be involved in the tumor progression; however its down-regulation is not considered a predictor factor of adverse outcome for these neoplasms [11].

The immunohistochemistry study showed that more than 10% of the neoplastic cells were positive for BAG1 and the basal cells of the neoplastic epithelium stained diffusely for bcl-2. Both BAG1 and bcl-2 play a role in the inhibition of apoptosis in endometrial carcinoma [12]. These data may suggest that anti-apoptotic stimuli are preponderant in this neoplasm. In endometrial carcinomas, bcl-2 expression does not provide prognostic information [13].

In this report, both HPV and p16 were negative, arguing against a direct role for HPV in the etiology of these neoplasms. This finding is in accordance with Lininger et al. (1997), who found HPV in only one of nine cases they analyzed [2].

Although the immunohistochemical findings of the present report are intriguing, no definite conclusions can be done based in a single case. Further studies with a larger patient series are needed to confirm the immunohistochemical findings in this type of tumor.

## Competing interests

The author(s) declare that they have no competing interests.

## Authors' contributions

ARS conceived the study and wrote the manuscript.
